# Expression Analysis of Four Peroxiredoxin Genes from *Tamarix hispida* in Response to Different Abiotic Stresses and Exogenous Abscisic Acid (ABA)

**DOI:** 10.3390/ijms13033751

**Published:** 2012-03-21

**Authors:** Caiqiu Gao, Kaimin Zhang, Guiyan Yang, Yucheng Wang

**Affiliations:** State Key Laboratory of Forest Genetics and Tree Breeding, Northeast Forestry University, 26 Hexing Road, 150040 Harbin, China; E-Mail: chwcaogcq@yahoo.com.cn; zkm831@yahoo.cn (K.Z.); zhgxuygy@yahoo.com.cn (G.Y.)

**Keywords:** *Prx* gene, gene expression, *Tamarix hispida*, abiotic stresses, ABA

## Abstract

Peroxiredoxins (Prxs) are a recently discovered family of antioxidant enzymes that catalyze the reduction of peroxides and alkyl peroxides. In this study, four *Prx* genes (named as *ThPrx*II, *ThPrx*IIE, *ThPrx*IIF, and *Th2CysPrx*) were cloned from *Tamarix hispida*. Their expression profiles in response to stimulus of NaCl, NaHCO_3_, PEG, CdCl_2_ and abscisic acid (ABA) in roots, stems and leaves of *T. hispida* were investigated using real-time RT-PCR. The results showed that the four *ThPrx*s were all expressed in roots, stems and leaves. Furthermore, the transcript levels of *ThPrx*IIE and *ThPrx*II were the lowest and the highest, respectively, in all tissue types. All the *ThPrx* genes were induced by both NaCl and NaHCO_3_ and reached their highest expression levels at the onset of stress in roots. Under PEG and CdCl_2_ stress, the expression patterns of these *ThPrxs* showed temporal and spatial specificity. The expressions of the *ThPrxs* were all differentially regulated by ABA, indicating that they are all involved in the ABA signaling pathway. These findings reveal a complex regulation of Prxs that is dependent on the type of Prx, tissue, and the signaling molecule. The divergence of the stress-dependent transcriptional regulation of the *ThPrx* gene family in *T. hispida* may provide an essential basis for the elucidation of Prx function in future work.

## 1. Introduction

Peroxiredoxins (Prxs), like catalase, superoxide dismutase, ascorbate peroxidase and glutathione peroxidase, are a group of prominent antioxidant enzymes in plants. They were first identified in 1996, when the Hv-1-CysPrx [1] and the Hv-2-CysPrx [2] were cloned from barley (*Hordeum vulgare*). Later, many Prxs were cloned and studied in other plants, such as *Arabidopsis* [3], rice (*Oryza sativa)* [4], liverwort (*Riccia fluitans*) [5], spinach (*Spinacia oleracea*) [6], poplar (*Populus* spp.) [7], tobacco (*Nicotiana tabacum*) [8] and winter rye (*Secale cereale*) [9]. Based on amino acid sequence similarities and specific structural features, mainly the number and position of conserved Cys residues, the Prx proteins have been grouped into four different families [10], namely 1-Cys Prx, 2-Cys Prx, type II Prx and type Q Prx. The members of 1-Cys Prx group have only one conserved Cys residue, and the 2-Cys Prx and type II Prx family members contain two conserved Cys residues, while the fourth group of Prx is the Q Prx type.

The function of Prxs in response to oxidative stresses has been studied in some depth. Prxs have also been reported to be involved in responses to other abiotic stresses, such as heat, cold, osmotic stress and high salinity [8,11–14]. Kim *et al.* [15] reported that the 2-Cys Prx (C2C-Prx1) from Chinese cabbage (*Brassica rapa*, subspecies *pekinensis* and *chinensis*) changed its protein structure from a low molecular weight (LMW) to a high molecular weight (HMW) complex against heat shock and oxidative stress. The 1-Cys Prx in Chinese cabbage also functions as a molecular chaperone under oxidative stress conditions [16]. Overexpression of At2-cys Prx in potato (*Solanum tuberosum)* enhanced tolerance to methyl viologen-mediated oxidative stress and high temperature [11]. Overexpression of PrxQ from *Suaeda salsa* (*SsPrxQ*) in *A. thaliana* increased tolerance to salt and cold stress [17]. The transgenic maize overexpressing *PrxQ* also showed the stress resistance against fungal disease and oxidative stress [8]. The transgenic tall fescue (*Festuca arundinacea*) overexpressing an Arabidopsis 2-Cys Prx is more tolerant against heat (42 °C) and methyl viologen (MV) stress than the control plants, and with less electrolyte leakage and thiobarbituric acid-reactive substances (TBARS) [18].

*Tamarix hispida* is a shrub or small tree growing mainly in arid and semi-arid regions, which exhibits tolerance to salt, drought and high temperature. This makes *T. hispida* an ideal model plant for the investigation of physiological and molecular mechanisms of responses to stresses in trees and for the cloning of a stress tolerance gene. In the present study, four *ThPrx* genes, including three type II Prxs and one 2-Cys Prx gene, were cloned from *T. hispida*. To better understand the possible roles of *ThPrx* genes in abiotic stress tolerance, the expression profiles of these four *ThPrx* genes in response to the application of salt (NaCl), salt- alkali (NaHCO_3_), drought (PEG), heavy metal (CdCl_2_) and abscisic acid (ABA) in the root, stem and leaf tissue of *T. hispida* were monitored by real-time RT-PCR.

## 2. Results

### 2.1. Cloning and Sequence Analysis of Four *ThPrx* Genes

**Figure 1:**
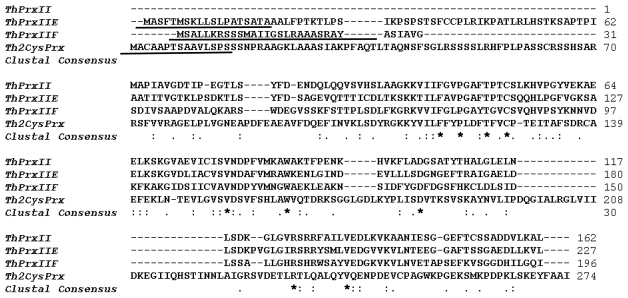
Multiple sequence alignments of the four ThPrx proteins from *T. hispida*. Signal prediction analysis showed the three ThPrxs contain signal peptides with 17–26 amino acids. The signal peptide of each ThPrx is underlined.

**Figure 2:**
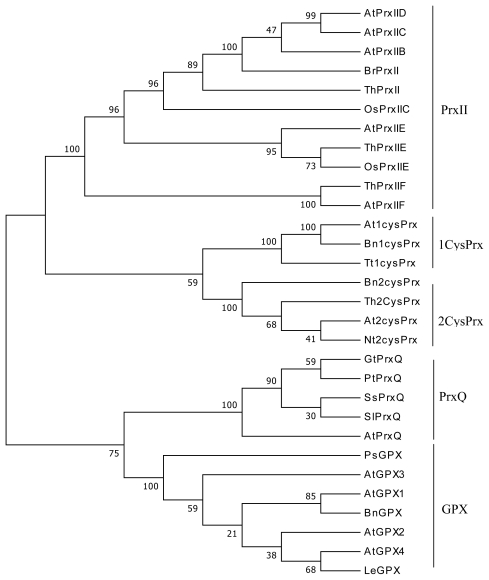
Phylogenetic tree of proteins homologous to Prx. Members of the four Prx proteins, including 2Cys-Prx, 1Cys-Prx, Prx-Q, and Type-II Prx, and in addition the Gpx family members were clustered with ClustalX. The first two letters correspond to the initials of the organism genus and species names. GenBank accession numbers are indicated: 1Cys-Prx: *Arabidopsis*, At1cysPrx (NP_175247); *Brassica napus*, Bn1cysPrx (AAF61460); *Triticum turgidum*, Tt1cysPrx (AAG50024). 2Cys-Prx: *Arabidopsis*, At2cysPrxA (NP_187769); *Brassica napus*, Bn2cysPrx (AAG30570); *Nicotiana tabacum*, Nt2cysPrx (CAC84143). Prx-Q: *Arabidopsis*, AtPrxQ (NP_189235); *Gentiana triflora*, GtPrxQ (BAD04985); *Populus trichocarpa* x *Populus deltoides*, PtPrxQ (AAS46230); *Suaeda salsa*, SsPrxQ (AAQ67661); *Sedum lineare*, SlPrxQ (BAA90524). Type-II Prx: *Arabidopsis*, AtPrxIIB (NP_176773); AtPrxIIC (NP_176772); AtPrxIID (NP_564763); AtPrxIIE (NP_190864); AtPrxIIF (NP_566268); *Oryza sativa*, OsPrxIIC (AAG40130); OsPrxIIE (BAA82377); *Brassica rapa*, BrPrxII (AF133302). GPX: *Arabidopsis*, AtGPX1 (NP_180080); AtGPX2 (NP_180715); AtGPX3 (NP_181863); AtGPX4 (NP_566128); *Pisum sativum*, PsGPX (sp|O24296); *Lycopersicon esculentum*, LeGPX (sp|O24031); *Brassica napus*, BnGPX (AAM12502).

Four *ThPrx* genes with complete open reading frames (ORFs) were identified from the six *T. hispida* libraries. The ORFs encoded deduced polypeptides of 162–274 amino acids, with a predicted molecular mass of 17.3–29.8 kDa and pI of 5.79–8.57 (Table 1). Except for *ThPrx*II, all *ThPrx* genes contain signal peptides with a length of 17–26 amino acids (Figure 1). Based on the number and position of conserved Cys residues, the Prxs were classified into four different types. According to this classification and the phylogenetic relationship among the Prx proteins, *ThPrx*II, *ThPrxIIE* and *ThPrxIIF* are type II Prx proteins, while *Th2CysPrx* belongs to 2-Cys Prx (Figure 2).

There were 14 ESTs representing the four unique *ThPrx* genes in the six libraries. The distribution of the 14 *ThPrx* ESTs was extremely heterogeneous in the libraries (Table 2). Among these ESTs, 12 ESTs were from the leaf libraries, and only 2 ESTs were identified in the root libraries. In addition, the number of ESTs representing different *ThPrx* genes in the library treated with NaHCO_3_ for 52 h increased one-fold compared with those in the library treated with NaHCO_3_ for 24 h, indicating that the transcription of *ThPrx* genes may be up-regulated by NaHCO_3_ stress in leaves.

### 2.2. Relative Expression Levels of Four *ThPrx* Genes in Roots, Stems and Leaves

Relative expression levels of the four *ThPrx* genes in *T. hispida* roots, stems and leaves under normal growing condition were studied by using real-time PCR. The transcription level of the gene Actin was assigned as 100, and the transcription levels of *ThPrx* genes were plotted relative to the *Actin* gene transcription level (Table 3). The results indicated that these *ThPrx* were expressed in all tissues including roots, stems and leaves. Among these *ThPrxs*, the transcription levels of *ThPrxIIE* were the lowest in roots, stems and leaves, while the transcription levels of *ThPrx*II were the highest in all tissues. Transcription levels of *Th2CysPrx* were the second highest except in roots, where the relative abundance of *Th2CysPrx* and *ThPrxIIF* were nearly similar.

### 2.3. Expression Profiles of *ThPrx* Genes in Response to Various Stresses

In order to study the relationship between the *ThPrx*s genes and stress response of *T. hispida*, the expression patterns of the four *ThPrx* genes in response to different abiotic stresses (NaCl, NaHCO_3_, PEG and CdCl_2_) and ABA application were investigated using real-time PCR.

#### 2.3.1. NaCl Stress

**Figure 3:**
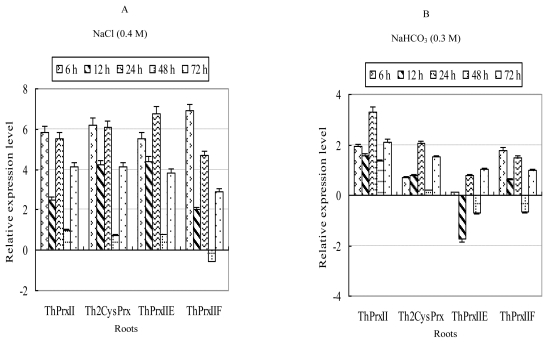

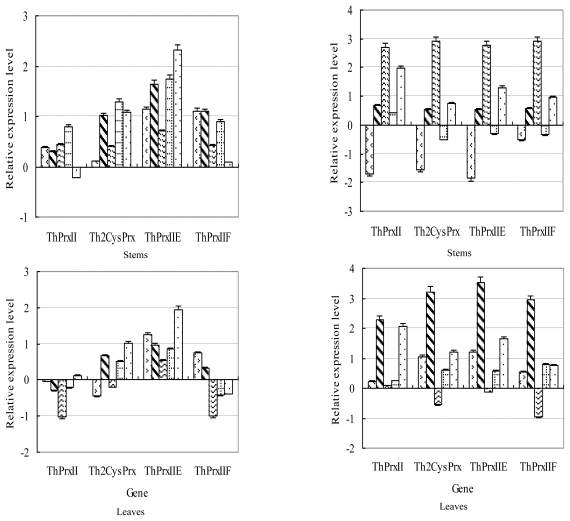
Transcription analysis of the four *ThPrxs* responding to NaCl and NaHCO_3_ stress in roots, stems and leaves. The relative transcription level = transcription level under stress treatment/transcription level under control condition (0 h). All relative transcription levels were log2-transformed. Error bars (SD) were obtained from nine replicates of the real-time PCR that included three biological replicates and each biological replicate contains three technical replicates. **A**: NaCl stress; **B**: NaHCO_3_ stress.

In roots, all four *ThPrx* genes with the exception of *ThPrxIIF* were highly induced by NaCl stress at all treatment times, with the highest transcription levels being induced more than 56.8-fold (Figure 3A). The transcription levels of *ThPrxII*, *ThPrxIIE* and *ThPrxIIF* reached their peak levels at an early time point (6 h). *Th2CysPrx* reached its highest transcription level at 24 h, with the second highest levels at 6 h. In stems, except for *ThPrxII* being down-regulated at 6 h, the other three *ThPrx*s genes were all induced throughout the treatment period. However, the induction rate in stems was lower than that in roots. The most highly induced gene was *ThPrxIIE*, which was induced 4.96-fold at 72 h of stress. In leaves, the expression patterns of the four *ThPrx*s genes were different. For instance, *ThPrx*II was down-regulated by NaCl stress. The transcription of *ThPrxIIF* was not altered at the early treatment stage and down-regulated at a later stage. *Th2CysPrx* was down-regulated at 6 and 24 h but was up-regulated at 12, 48 and 72 h. *ThPrxIIE* was up-regulated at all time points.

#### 2.3.2. NaHCO_3_ Stress

In roots, all four *ThPrx* genes were up-regulated at most time points; especially *ThPrxII* and *Th2CysPrx* which were induced throughout the entire treatment period. In stems, all genes were down-regulated at an early time point (6 h), followed by up regulation. They all reached their peak expression levels at 24 h of stress. In leaves, the four *ThPrx* genes were induced at an early time point, and reached their highest expression levels at 12 h. All genes were down-regulated at 24 h. Furthermore, the expression levels were equal to or lower than their levels at 0 h (Figure 3B).

#### 2.3.3. PEG Stress

**Figure 4:**
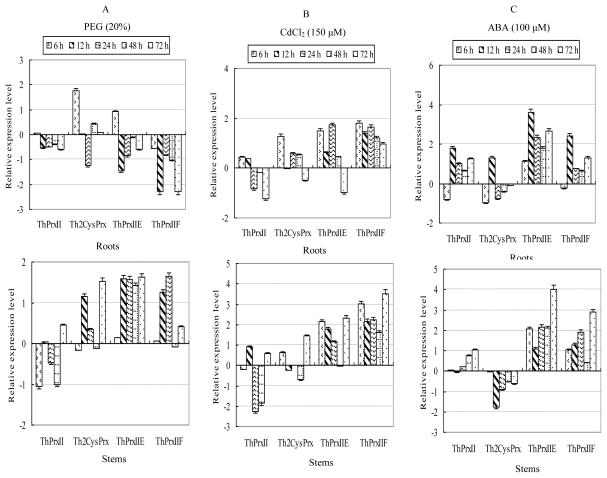

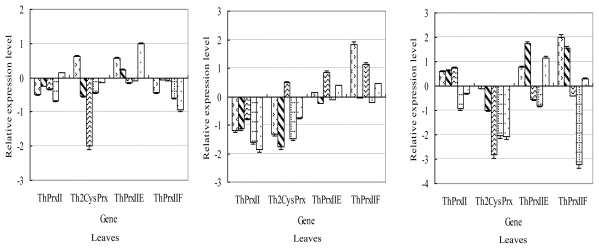
Expression analysis of the four *ThPrxs* responding to application of abscisic acid (ABA), and treatments of PEG and CdCl_2_ in roots, stems and leaves. The relative transcription level = transcription level under stress treatment/transcription level under control condition (0 h). All relative transcription levels were log2-transformed. Error bars (SD) were obtained from nine replicates of the real-time PCR that included three biological replicates and each biological replicate contains three technical replicates. **A**: PEG stress; **B**: CdCl_2_ stress; **C**: ABA treatment.

In roots, the transcription levels of *ThPrxII*, *ThPrxIIE* and *ThPrxIIF* were generally decreased, while *Th2CysPrx* at most time points was up-regulated. In stems, *ThPrxII* was down-regulated, while the other three *ThPrx*s were all up-regulated. In leaves, all four *ThPrx* gene transcriptions were down-regulated (Figure 4A).

#### 2.3.4. CdCl_2_ Stress

The transcription levels of the four *ThPrx* genes were divided into two distinct groups. One group contained *ThPrxIIE* and *ThPrxIIF*, and they shared similar expression patterns, while *ThPrxII* and *Th2CysPrx* constituted the other group. In roots, *ThPrxIIE* and *ThPrxIIF*, were up-regulated during the CdCl_2_ stress period, except at 72 h. In contrast, *ThPrxII* and *Th2CysPrx* were down-regulated for at least three time points. The transcription level of *ThPrx*1 decreased by 58% at 72 h, when compared with levels at 0 h. In stems, *ThPrxIIE* and *ThPrxIIF* were up-regulated at all stress times. *ThPrxII* and *Th2CysPrx* levels showed alternating up- and down-regulation patterns. In leaves, the expression pattern was opposite to stems. *ThPrxII* and *Th2CysPrx* were down-regulated, while *ThPrxIIE* and *ThPrxIIF* levels showed alternating up- and down-regulation patterns (Figure 4B).

#### 2.3.5. ABA Application

The RT-PCR results demonstrated that the four *ThPrx* genes shared similar expression patterns in roots, stems and leaves under the regulation of ABA. *ThPrxII*, *ThPrxIIE* and *ThPrxIIF* were up-regulated at most time points. In contrast, *Th2CysPrx* transcription was down-regulated, especially in stems and leaves (Figure 4C).

## 3. Discussion

The *Prx* gene family is ubiquitously distributed in all organisms from bacteria to higher plants. It is a small gene family with only 10 genes in *Arabidopsis* and 11 genes in rice. In this study, we cloned four *Prx* genes with complete ORFs from *T. hispida*, including three type II Prxs and one 2-Cys Prx. The transcription levels of the four *ThPrx* genes were notably different under normal growth conditions (Table 4). *Th2CysPrx*, as the other type Prx gene, was also abundant in stems and leaves. Especially in leaves, where its abundance was slightly lower than that of *ThPrxII*. Muthuramalingam *et al.* [19] confirmed that the 2-Cys peroxiredoxin can act as a regulatory hub in the chloroplast; therefore, *Th2CysPrx* may also act as a key peroxiredoxin in chloroplast.

Here, we have shown that these four *ThPrx* genes are induced by at least two types of abiotic stresses, indicating that all four *ThPrx* genes may play roles in abiotic stress response of *T. hispida*. Previous studies demonstrated that the transcription of *Prx* genes are in response to different kinds of stresses, such as low or high light, salinity, heavy metals, nutrient deprivation, temperature extremes and chemical effectors [20]. For example, the transcript levels of the *1-Cys Prx* gene from *Xerophyta viscosa* Baker increased when subjected to dehydration, heat (42 °C), high light intensity (1500 μmol photons m^−2^·s^−1^), NaCl (100 mM) and 100 μM ABA [21]. Finkemeier *et al.* [12] observed that the transcripts of *AtPrxII F* were increased in roots of *Arabidopsis* under CdCl_2_ stress. *AtPrxIIC* transcript levels responded strongly during oxidative stress [22]. In pea leaves, *PrxII F* protein accumulates upon cold and heavy-metal treatment [23].

Interestingly, under drought (20% PEG) and heavy metal (CdCl_2_) stress conditions, the four *ThPrx* genes displayed different expression patterns. Furthermore, they showed different responses to different stress types, times and organ-specific variations. At the same time, all four *ThPrx* genes were induced by both salt (NaCl) and salt-alkali (NaHCO_3_) stress. In roots, all four genes reached their highest transcription levels at an early NaCl stress stage (6 h), suggesting that the transcripts of these *ThPrx* genes are triggered rapidly under stress conditions. Prx proteins are found to play a central role in ROS detoxification [23]; therefore, these *ThPrxs* that are all highly induced by stresses may also play important roles in ROS detoxification in cells under salt stress.

Dietz *et al.* [20] summarized and demonstrated that *Prx* transcript regulation varied, depending on the type of *Prx*, plant species, stress intensity, and developmental state. Consistent with these results, *Th2CysPrx*, the unique 2-Cys Prx gene among the studied four *ThPrx*s, showed the most peculiar expression pattern under different stress conditions and application of ABA. Its transcription was stimulated upon an increase in the roots after PEG stress and decreased in all organs by ABA application. The divergence of the stress-dependent expressional regulation among the four *ThPrx* genes in *T. hispida* suggest that, compared with the other *ThPrx* genes, *Th2CysPrx* may play different roles in resistance to stress or may be involved in different signaling transduction processes. Until now, expressional regulation of *Prx* on promoter and signaling levels has only been investigated for *At1-CysPrx* and *At2-CysPrx* [24–25]. Therefore it is essential to study the expressional regulation of *ThPrx*s in response to stress tolerances on the promoter and signaling levels in the future.

## 4. Experimental Section

### 4.1. Plant Materials and Stress Treatments

Seedlings of *T. hispida* were grown in pots containing a mixture of turf peat and sand (2:1 *v*/*v*) in a greenhouse under controlled conditions of 70–75% relative humidity, light/dark cycles of 14/10 h with lights on at 7.00 AM, and maintaining a temperature of 24 °C. Two month-old seedlings were used for experimental analysis. To induce abiotic stresses, the seedlings were watered into their roots with solutions of 0.4 M NaCl, 0.3 M NaHCO_3_, 20% (*w*/*v*) PEG6000 and 150 μM CdCl_2_ for 0, 6, 12, 24, 48 and 72 h, respectively. For ABA treatment, the seedlings were exposed to 100 μM ABA solution (0.1% *v*/*v* ethanol). The ABA solution was watered into the roots of seedlings. For the control, the seedlings were exposed with the same volume of water containing only the same concentration of ethanol without ABA. The leaves, stems and roots from at least 24 seedlings were collected and pooled after various stress time points (0, 6, 12, 24, 48 and 72 h), immediately frozen in liquid nitrogen and stored at −80 °C until further use. Three samples from each treatment were prepared for real-time PCR biological repeats.

### 4.2. Cloning and Identification of 4 *ThPrx* Genes

Six cDNA libraries were constructed including three samples from *T. hispida* leaves [26] and three from *T. hispida* roots [27]. In total, 17,173 ESTs were obtained from the six libraries. The ESTs were assembled into singletons and contigs, with the parameters set at 40 bp overlap and 95% identity using the CAP3 assembly program. The functional annotation of ESTs was performed using BLASTX and BLASTN against the non-redundant (NR) NCBI database. The ESTs representing the four *ThPrx* genes were identified according to their functional annotations. The library clones containing the *Prxs* genes were further sequenced from both sides to confirm their sequences and to generate complete sequence data.

### 4.3. Sequence Alignments and Phylogenetic Analysis

All four ThPrx protein sequences were aligned by ClustalX. The Prx proteins from *T. hispida* and other plants were subjected to phylogenetic analysis by conducting a phylogenetic tree reconstruction employing the neighbor-joining (NJ) method in ClustalX. Furthermore, the classification of the four *ThPrx* genes was carried out according to the classification and designation method of Bréhélin *et al.*[9]. Signal peptide predictions for the four ThPrx proteins were performed using the Signal peptide tool (http://www.cbs.dtu.dk/services/SignalP/). Molecular weight (MW) and isoelectric point (pI) predictions for every deduced ThPrxs were carried out by the Compute pI/Mw tool (http://www.expasy.org/tools/protparam.html).

### 4.4. RNA Extraction and Reverse Transcription (RT)

Total RNA was isolated from leaves, roots or stems using a CTAB method and digested with DNase I (Promega, Madison, WI, USA) to remove any DNA residue. Approximately 0.5 μg of total RNA was reversely transcribed (RT) into cDNA using an oligodeoxythymidine primer and six random primers in a final reaction volume of 10 μL following the PrimeScript™ RT reagent Kit protocol (TaKaRa). The synthesized cDNAs were diluted to 100 μL with sterile water and used as the template for real-time RT-PCR.

### 4.5. Real-time Quantitative RT-PCR

Real-time RT-PCR was performed using an MJ Opticon™^2^ machine (Biorad, Hercules, CA, USA). The genes of alpha tubulin (FJ618518), beta tubulin (FJ618519) and beta actin (FJ618517) were chosen as internal controls (reference gene) to normalize the amount of total RNA present in each reaction. The 20 μL reaction mixture contained 10 μL of SYBR-Green Real-time PCR Master Mix (Toyobo), 0.5 μM of specific primers for *ThPrx* genes (Table 4) or reference genes, and 2 μL cDNA template (equivalent to 100 ng of total RNA).

The amplification was completed with the following cycling parameters: 94 °C for 30 s, followed by 45 cycles at 94 °C for 12 s, 60 °C for 30 s, 72 °C for 40 s and 1 s at 81 °C for plate reading. A melting curve was generated for each sample at the end of each run to assess the purity of the amplified products. For each sample, reactions were carried out in triplicate to ensure the reproducibility of the results. The gene transcription levels of the clones were calculated from the threshold cycle according to 2^−ΔΔCt^ [28].

## 5. Conclusions

In conclusion, we cloned four *ThPrx* genes with complete ORFs from *T. hispida*, including three type II Prxs and one 2-Cys Prx. Expression analysis showed that these four *ThPrx* genes are all expressed in the roots, stem and leaves, suggesting that they play roles in all of these tissues. In addition, these four genes are all associated with abiotic stress responses and are involved in the ABA signaling pathway. Consequently, some of these *ThPrx* genes may have potential for use in the genetic improvement of abiotic stress tolerance in plants.

## Figures and Tables

**Table 1:** Characteristics of the four *ThPrxs* from *T. hispida*.

Gene	GenBank accession number	Type	Deduced number of amino acid	Isoelectric point	Molecular mass (kDa)
*ThPrxII*	JQ341201	type II peroxiredoxin	162	5.79	17.3
*Th2CysPrx*	JQ341202	2Cys peroxiredoxin	274	6.9	29.8
*ThPrxIIE*	JQ341203	type II peroxiredoxin	227	8.57	24.0
*ThPrxIIF*	JQ341204	type II peroxiredoxin	196	8.37	21.0

**Table 2:** The distribution of *ThPrx* ESTs among the *T. hispida* cDNA libraries.

Gene	Library					

	Root libraries			Leaf libraries		

	0 h	24 h	48 h	0 h	24 h	52 h
*ThPrxII*	1	0	0	0	1	2
*Th2CysPrx*	0	0	0	0	0	1
*ThPrxIIE*	0	0	0	2	1	0
*ThPrxIIF*	0	0	1	1	1	3

Total	1	0	1	3	3	6

**Table 3:** Relative abundance of the four *ThPrx*s in different tissues of *T. hispida.* The transcription levels of the four *ThPrx* genes were plotted relative to the expression of *Actin* gene, and the transcription levels of *Actin* gene in root, stem and leaf were all assigned as 100.

Gene	Relative abundance		

	Roots	Stems	Leaves
*ThPrxII*	83.4	155.6	226.9
*Th2CysPrx*	17.8	42.3	187.9
*ThPrxIIE*	5.6	6.3	30.4
*ThPrxIIF*	20.1	15.0	38.4
*Actin*	100	100	100

**Table 4:** Primer sequences used for quantitative RT-PCR analysis

Gene	Forward Primers (5′–3′)	Reverse Primers (5′–3′)
*ThPrxII*	TCAGCAGGTTTCAGTTCACT	CAGAGCCATCAGCAAGGA
*Th2CysPrx*	GAGAAGGCTTGGACTGAG	GAGGAACGGCAAGATGAG
*ThPrxIIE*	CCCTCTCCTATTTTGACTCC	TCAGCAGCAGGACTTCATC
*ThPrxIIF*	CTTCCTCATCGGAAATATGTCG	AACAAACACCTGTGTACGCACC
*Actin*	AAACAATGGCTGATGCTG	ACAATACCGTGCTCAATAGG
*α-tubulin*	CACCCACCGTTGTTCCAG	ACCGTCGTCATCTTCACC
*β-tubulin*	GGAAGCCATAGAAAGACC	CAACAAATGTGGGATGCT
